# Spontaneous Atraumatic Subdural Hematoma Related to Methamphetamine Use

**DOI:** 10.7759/cureus.18383

**Published:** 2021-09-29

**Authors:** Anthony Nguyen, Laura Reed, Samuel R Daly, Kristin Keith, David Garrett

**Affiliations:** 1 Neurosurgery, Baylor Scott & White Health, Temple, USA

**Keywords:** neurological surgery, methamphetamine, burr holes, trephination, subdural hematoma, neurosurgery

## Abstract

There are multiple risk factors associated with spontaneous subdural hematoma (SDH), including substance abuse, hypertension, vascular abnormalities, and neoplasms. The illicit drugs typically cited as risk factors for spontaneous SDH are alcohol and cocaine. We report a rare case of spontaneous, significant SDH associated exclusively with methamphetamine. Although it is unclear whether the underlying pathophysiology involves vasculitis, sympathomimetic-induced hypertension, or a combination of both, this case further illustrates the risks of methamphetamine abuse.

## Introduction

Subdural hematomas (SDHs) are an accumulation of blood products in the subdural space, often caused by damage to the bridging veins [1]. Most frequently, SDHs in adult patients are caused by falls, assaults, and high-speed accidents [2,3]. Drug abuse, specifically alcohol, is implicated in several SDH cases [2,3]. The treatment of SDH varies depending on the chronicity of the clot and can also be influenced by whether there are mixed densities on computed tomography (CT), which suggests an acute-on-chronic SDH [4-6]. Treatment options include burr holes, craniotomy or craniectomy with or without placement of a subdural drain, and middle meningeal artery embolization [4,7].

Although trauma is commonly implicated in SDHs, spontaneous etiologies have also been reported in the literature, which are estimated to contribute to 3%-5% of SDH cases [8,9]. Spontaneous SDHs are often due to vascular anomalies such as aneurysms, fistulas, or arteriovenous malformations; however, it can also be caused by medical conditions such as hypertension and malignancy [8]. We report here the case of a male in his 30s who was found to have a spontaneous acute-on-chronic subdural hematoma associated with methamphetamine use.

## Case presentation

**Figure 1:**
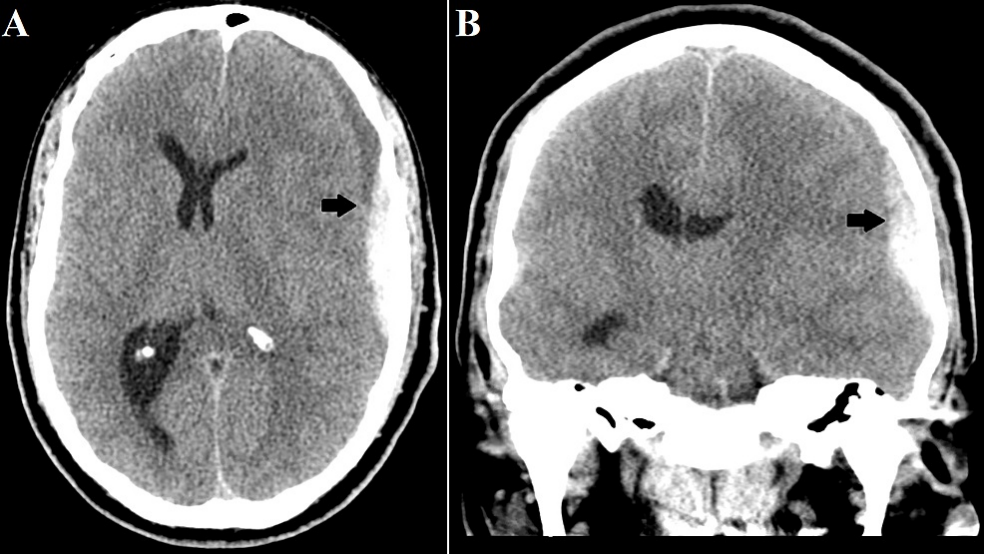
Preoperative computed tomography scan of the patient’s head demonstrating a 10 mm heterogeneously dense subdural hematoma (black arrow) with 10 mm of midline shift in axial (A) and coronal (B) planes.

A 39-year-old male presented to an emergency department with a worsening headache of one-week duration. He was found on CT to have an acute SDH with almost 10 mm of midline shift and was therefore transferred to our level I trauma center. He denied any traumatic head injury when questioned extensively and stated that the only notable event was him forcefully blowing his nose the day his headache started. He denied any past medical history or use of antiplatelets or anticoagulants but admitted to daily tobacco use and frequent methamphetamine use. He denied alcohol use. He denied any other symptoms. He was mildly hypertensive with systolic blood pressure between 140 and 169 mmHg. On physical examination, he was neurologically non-focal without a pronator drift, his Glasgow Coma Scale (GCS) score was 15, and he did not have any findings suggesting trauma. His urine drug screen was positive for methamphetamine. An interval head CT was obtained (Figure 1) with the addition of angiography and venography, given the lack of a history of a traumatic event. These were negative for vascular anomalies. A surgical evacuation was offered to the patient, and he provided informed consent for burr holes.

**Figure 2:**
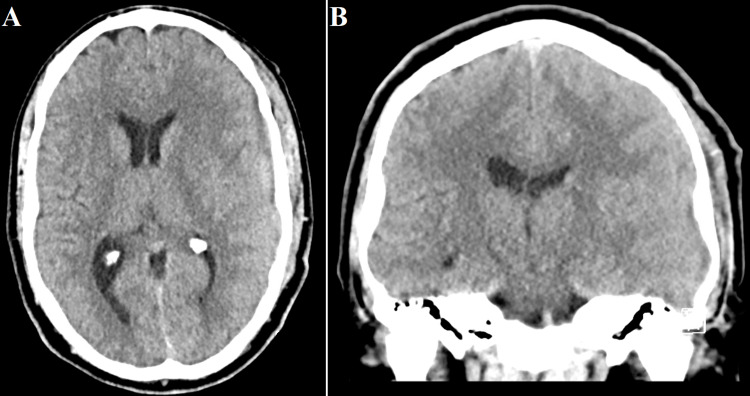
Postoperative computed tomography scan of the patient’s head performed several days after surgery demonstrating significant reduction of the subdural hematoma and the associated midline shift in axial (A) and coronal (B) planes.

The patient was taken to the operating room for SDH evacuation, and two left-sided burr holes were created in a standard fashion. The anterior burr hole revealed hemosiderin-stained dura mater, and upon incision of the dura, thick subdural membranes were encountered. No acute bleeding could be found, and the blood products were copiously irrigated. A subdural drain was placed, and the incisions were closed. The patient was extubated and transferred to the intensive care unit, and a postoperative head CT demonstrated improvement of his midline shift. His subdural drain was removed on postoperative day 1. A final CT performed two days after drain removal (Figure 2) showed significant improvement. He remained GCS 15 without neurologic deficit and was discharged on postoperative day 6. The patient’s family members took him to their home in another state, and he was lost to follow-up.

## Discussion

We describe here the case of a patient developing a spontaneous, nontraumatic SDH possibly associated with methamphetamine use. The imaging and intraoperative findings were suggestive of an acute-on-chronic SDH. Although the nose-blowing event may have precipitated the acute component, this was not a traumatic injury by any means. The patient had also likely sustained another spontaneous SDH in the past, explaining the thick membranes found intraoperatively, which had increased his risk for further SDHs.

Previously, spontaneous SDH associated with cocaine use has been described in the literature with the hypothesized mechanism being acute hypertension during cocaine use and chronically weakened blood vessels [10]. Methamphetamine has been implicated in cerebrovascular disease and is also a sympathomimetic like cocaine [11]. The search terms “subdural” and “amphetamine" were used in the PubMed database search. Although it has been described in postmortem series as being associated with thin SDHs, methamphetamine use causing a significant intracranial SDH has only been described in the literature once [12,13]. Thus, we report here the second case of a significant spontaneous intracranial SDH caused by methamphetamine use. However, there are a few notable differences between the aforementioned case and the case of our patient. First, the patient described in the case by Nagele et al. sustained a single acute SDH, whereas our patient had an acute-on-chronic SDH, suggesting that our patient had at least two methamphetamine-related subdural bleeds. Second, their patient had a history of pulmonary embolism and was only 27 years old, suggesting the possibility of an underlying hematologic condition. There was also no mention of whether the patient was on any anticoagulation. Their patient also utilized methamphetamine in combination with alcohol and tetrahydrocannabinol. Animal studies have demonstrated that the coadministration of methamphetamine and alcohol impairs antioxidant enzymes and increases serum concentrations of methamphetamine and amphetamine [14]. The combination may thus theoretically increase the risk of vasculitis or transient hypertension.

There are some limitations to note of this study. First, this is a case report of a single patient. Although he denied traumatic injury and the physical examination findings were not revealing a traumatic injury, it is not possible to state with absolute certainty that the patient was entirely honest regarding atraumatic onset. However, as the patient was forthcoming regarding methamphetamine usage, there is no particular reason to doubt his provided history. Prospective studies of the effects of methamphetamine on cerebral vasculature would help provide further insight into whether methamphetamine-related SDH is caused by hypertension, vasculitis, or a combination of both or whether it can be caused by either mechanism.

## Conclusions

SDHs are a clinical entity commonly encountered by neurosurgeons. When the SDH size or symptoms are significant, surgical intervention may be indicated. However, spontaneous SDHs are a rare clinical entity and can be due to several possible etiologies, ranging from neoplasm to hypertension, which may be secondary to drug use. The clinician should be aware that drug-induced hypertension or vasculitis can cause spontaneous subdural hematomas even in young, otherwise healthy patients. Although cocaine is more commonly implicated in drug-related SDHs, methamphetamine can also result in atraumatic SDHs.
